# Synergistic Interaction and Binding Efficiency of Tetracaine Hydrochloride (Anesthetic Drug) with Anionic Surfactants in the Presence of NaCl Solution Using Surface Tension and UV–Visible Spectroscopic Methods

**DOI:** 10.3390/gels8040234

**Published:** 2022-04-11

**Authors:** Naved Azum, Malik Abdul Rub, Anish Khan, Maha M. Alotaibi, Abdullah M. Asiri

**Affiliations:** 1 Center of Excellence for Advanced Materials Research, King Abdulaziz University, Jeddah 21589, Saudi Arabia; nhassan2@kau.edu.sa (N.A.); akrkhan@kau.edu.sa (A.K.); aasiri2@kau.edu.sa (A.M.A.); 2 Chemistry Department, Faculty of Science, King Abdulaziz University, Jeddah 21589, Saudi Arabia; mmsalotaibi@kau.edu.sa

**Keywords:** tetracaine hydrochloride, sodium dodecyl sulfate, sodium lauroyl sarcosine, drug–surfactant mixed micelle, synergistic interaction

## Abstract

Surfactants are ubiquitous materials that are used in diverse formulations of various products. For instance, they improve the formulation of gel by improving its wetting and rheological properties. Here, we describe the effects of anionic surfactants on an anesthetic drug, tetracaine hydrochloride (TCH), in NaCl solution with tensiometry and UV–visible techniques. Various micellar, interfacial, and thermodynamic parameters were estimated. The outputs were examined by using different theoretical models to attain a profound knowledge of drug–surfactant mixtures. The presence of attractive interactions among drug and surfactant monomers (synergism) in mixed micelle was inferred. However, it was found that sodium dodecyl sulfate (SDS) showed greater interactions with the drug in comparison to sodium lauryl sarcosine (SLS). The binding of the drug with surfactants was monitored with a spectroscopic technique (UV–visible spectra). The results of this study could help optimize the compositions of these mixed aggregates and find the synergism between monomers of different used amphiphiles.

## 1. Introduction

It is often observed that the surfactant mixtures (e.g., surfactant–co-polymer, surfactant–drug, and surfactant–surfactant) exhibit better performance than single surfactants [1,2,3,4,5,6]. It is also common to use mixtures of surfactants and polymers to formulate gels that are used in drug-dosage forms to improve their properties or to improve their physical stability [7]. The anionic surfactant used in this study, sodium dodecyl sulfate, has been used to synthesize nanogels [8]. SDS has shown better activity in the formation of microgels based on poly(N-isopropylacrylamide) [8]. The synergistic or antagonistic effects of binary mixtures are produced by attraction or repulsion between surfactant monomers. Synergism is observed when the molecular interaction between the monomers of a mixture is greater than before mixing. The strength of synergism between different types of surfactants follows the order of anionic–cationic > nonionic–ionic > ionic–ionic > nonionic–nonionic. The interaction between oppositely charged head groups and the hydrophobic interaction between chains of amphiphiles are the two main factors that are responsible for strong synergistic effects inside cationic–anionic mixtures [9,10,11]. Ionic–anionic mixtures become turbid (precipitation) at some mole fractions, producing lamellar phases and rod-like morphologies.

A lesser water solubility and the dissolution characteristics of a drug usually limit its bioavailability and therapeutic efficacy. The poor water-solubility of drugs may also lead to disappointing and inconstant ingesting, which aggravates the complications of bioavailability and scarcity in the delivery of drugs. In addition, excessive dosages of drugs cause side effects such as vomiting, nausea, dizziness, and fatigue [12,13]. The development of increasing water solubility and improvements in encapsulation efficiency can enhance absorption, enhance bioavailability, and lower the required therapeutic dose [1,14,15]. Researchers have often studied different ways to increase solubilities, such as using small drug carriers, preparing nanoparticles, and using self-emulsifying formulations or amorphous formulations based on water-soluble polymers. A surfactant is a most-capable drug transporter in biomedical applications since it can be easily fabricated into different formulations such as micelles, hydrogels, and nanoparticles to enclose bioactive agents at several points of hydrophobicity [16,17,18,19]. Surfactants are polar molecules and contain both hydrophilic and hydrophobic components orientated at the surface to diminish the surface tension of water [20,21,22]. A micelle will only form when the concentration of the amphiphile is higher than a specific concentration (called the critical micelle concentration or cmc) that can be determined using diverse methods (surface tension, conductometry, fluorometry, UV–visible spectroscopy, cyclic voltammetry, and isothermal calorimetry) [23,24,25,26]. A valuable feature of these molecules is their cmc value. The cmc value depends on various aspects such as ionic strength, temperature, and the existence of additional compounds in the solution. Most chemical industries utilize surfactants, e.g., as pharmaceuticals, corrosion inhibitors, detergents, paints, and cosmetics [27,28,29,30,31].

**Figure 1:**
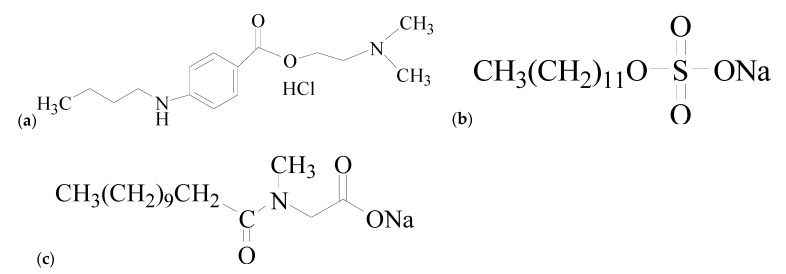
Chemical structures of (**a**) tetracaine hydrochloride (TCH), (**b**) sodium dodecyl sulfate (SDS), and (**c**) sodium lauryl sarcosine (SLS).

Certain types of drugs, such as antidepressants, anticholinergics, antihistamines, and local anesthetics, are amphiphilic; they have surfactant-like properties and form micelles [32,33,34,35]. Invariably, their therapeutic activity is determined by how they interact with surfactants. Depending on their interactions in solution, any drug can be made more active. The mixed systems of many amphiphilic drugs have also been researched by our group using different techniques with different amphiphiles [36,37,38,39,40,41,42,43,44,45]. Tetracaine hydrochloride, TCH (Figure 1), is an amphiphilic compound that also possesses colloidal properties and is one of the most used local anesthetic drugs. It is used for stopping pain during surgery and eye infections. Since tetracaine is a poorly water-soluble compound, it is usually formulated as tetracaine hydrochloride. It has been hypothesized that the +ve charge on the drug, which is the functional component, interacts with the Na^+^ channels on neuronal membranes and stops the transmission of the pain sensation along the nerve [46,47]. Furthermore, the cationic form provides an amphiphilic structure to such a drug, so it can be classified as a cationic tension-active molecule. Therefore, a TCH-like cationic surfactant undergoes an abrupt change above a critical concentration (cmc) and the Krafft temperature. The aqueous dissolution of tetracaine follows the same principle as all ionic surfactants (in that it is governed by both solubility and micellization). As a result, the nature of the surfactant, its counter ions, concentration, and temperature all affect the process. As the use of high concentrations of local anesthetic in spinal anesthesia is known to occasionally result in the sudden death of patients, it is important to understand how the micellization process occurs and what its phase diagram looks like.

In this work, surface tension and UV–visible measurements were carried out to examine the effects of anionic surfactants on a cationic drug. To the best of our knowledge, the mixed micellization of tetracaine hydrochloride (TCH) with sodium lauroyl sarcosine (SLS) and sodium dodecyl sulfate (SDS) in the presence of sodium chloride (NaCl) has not been previously described. Different theoretical approaches of mixed micellization (such as those by Clint, Rubingh, Rodenas, Rosen, and Motomura) were utilized to investigate the interactions of TCH + SDS/SLS mixtures. Various interfacial, micellization, and energetic parameters were analyzed. The output of this work can support the search for a surfactant-based carrier for drug delivery.

## 2. Result and Discussion

**Figure 2:**
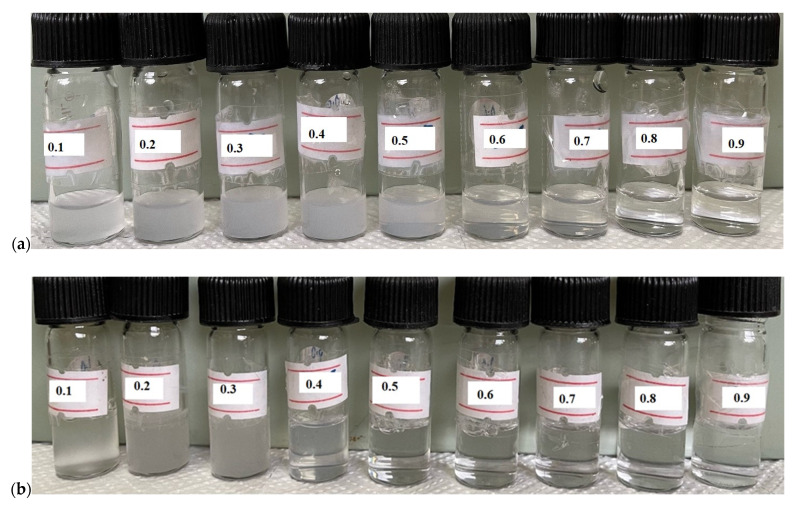
The physical appearance of TCH + SDS/SLS mixtures at different compositions: (**a**) SDS + TCH and (**b**) SLS + TCH.

**Figure 3:**
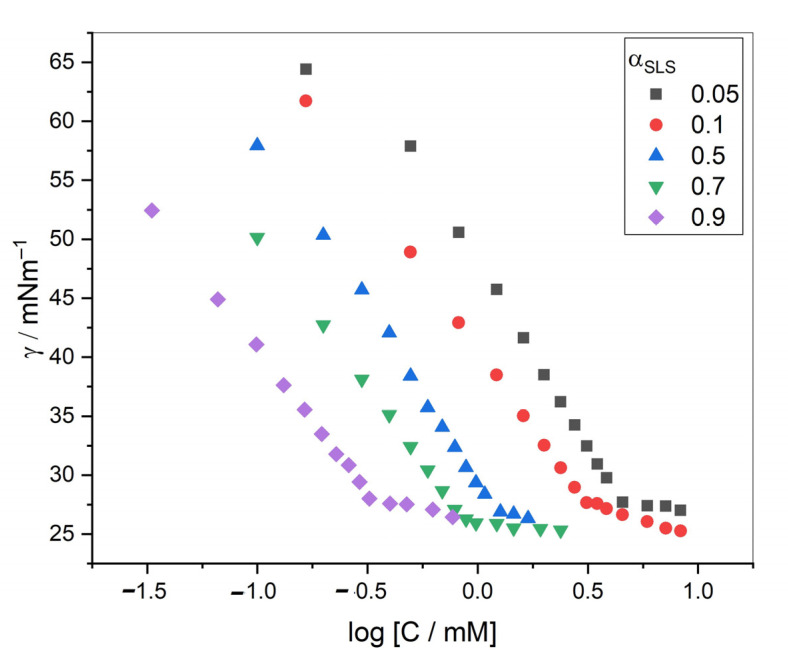
Graph of surface tension versus log molar concentration for SLS + TCH mixed systems.

The stock solutions of numerous mole fractions ($\alpha_{1}$) of component 1 (SDS/SLS) from 0 to 1 were prepared. As shown in Figure 2, the solution was turbid at some mole fractions (which barred the experiment), and we selected the mole fractions where no turbidity was observed. The surface tension (ST) measurements were used to estimate the cmc values of pure and binary mixtures of drugs and surfactants. Measurements of surface tension are widely used to provide authentic cmc values for all types of surfactants (cationic, anionic, and non-ionic). Illustrative ST graphs for the mixtures at different mole fractions of SLS in the presence of 100 mM NaCl at 298.15 K are displayed in Figure 3. The cmc values acquired via surface tension are listed in Table 1. As the surfactant molecules were mixed, a complex, which was more deeply adsorbed at the surface than single amphiphiles, was formed, thus suggesting an enhanced surface activity. The cmc values of single and mixed amphiphiles could be evaluated by the intersection of the linear fitting of the points (Figure 3). The cmc value of TCH was found to be 79.43 mM, which was lower than the values published by Miller et al. [48], who reported a value of nearly 100 mM without any salt. The cmc values of both employed surfactants in the existence of salt were also found to be less than those with a lack of salt. The values of cmc for currently employed surfactants in the presence of NaCl were in good agreement with the literature [49,50]. The obtained value of cmc for SDS in the presence of 100 mM NaCl was much lower than the cmc value computed by Thapa et al. [51] in an aqueous solution. When NaCl was added to the drug solution, the electrical atmosphere changed. The charge between the head group in the cationic drug became neutralized. Micelles could be formed at much lower concentrations in pure water because of the reduced electrostatic repulsion among the polar head groups. The cmc values for all mixtures unified in the center of two single amphiphiles, suggesting that the micellization of a drug was preferred in the company of surfactants. The observed decline in the cmc values of the mixture was due to the enrichment in the hydrophobic interaction among drugs and surfactants.

The whole study can be divided into two parts: (A) interactions of drugs with surfactants in the solution and (B) interactions of drugs with surfactants at the surface.

### 2.1. Interactions of Drug with the Surfactants in the Mixed Micelle

Using Rubingh’s regular solution theory (RST) for mixtures of amphiphiles [52], the cmc of a mixed system (cmc*) can be calculated via Equation (1):

(1) $$\frac{1}{cmc^{*}}=\frac{\alpha_{1}}{f_{1}cmc_{1}}+\frac{\alpha_{2}}{f_{2}cmc_{2}}\;$$

where *f*_1_ and *f*_2_ are the activity coefficients of the surfactant (SDS/SLS) and drug in mixed micelles, respectively, and $\alpha_{1}$ represents the mole fraction of surfactant (SDS/SLS) in the total mixed solution. The cmc values of surfactants and drugs are $cmc_{1}$ and $cmc_{2}$, respectively. *f*_1_ = *f*_2_ = 1 if we assume ideal behavior, so Equation (1) becomes:

(2) $$\frac{1}{cmc^{*}}=\frac{\alpha_{1}}{cmc_{1}}+\frac{\alpha_{2}}{cmc_{2}}\;$$

**Figure 4:**
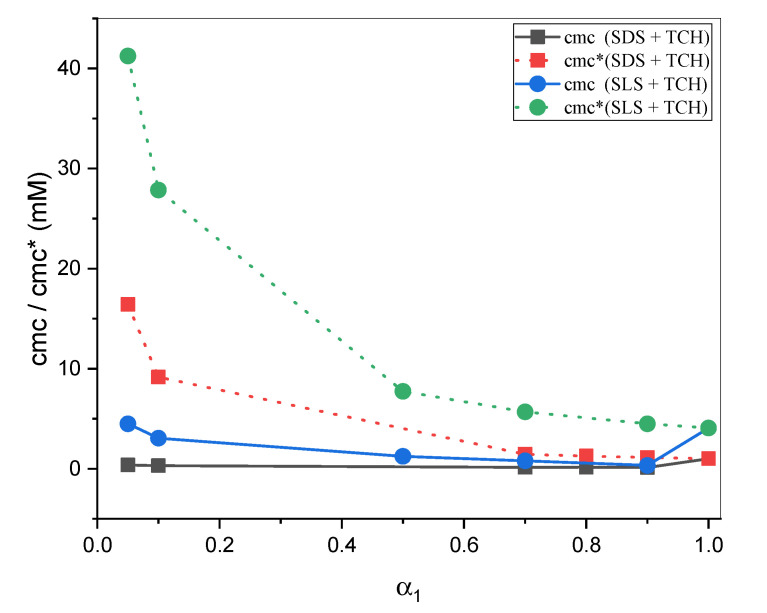
Experimentally determined critical micelle concentration (*cmc*) and ideal critical micelle concentration (cmc*) against mole fraction of surfactants (SDS/SLS) in mixed systems at 298.15 K.

Equation (2) was proposed by Clint [53]. Using the Clint equation, we could judge the ideality or non-ideality of a mixed system. Figure 4 displays a plot of *cmc* (experimentally determined)/cmc* (calculated with Equation (2)) vs. *α*_1_ (SDS/SLS). The *cmc* values of both mixtures were decreased with increases in the α_1_. According to one possible explanation, the mixture was more favorable than expected under an ideal condition because of the interactions among hydrophobic chains of amphiphiles.

In contrast, for non-ideal mixtures, a new theory has been established and is referred to as the Rubingh model [52]. The Rubingh model uses RST to relate the activity coefficients of components with micellar mole fractions of component 1 as follows:

(3) $$f_{1}^{Rub}=exp\left[\beta^{Rub}{\left(1-X_{1}^{Rub}\right)}^{2}\right]$$

(4) $$f_{2}^{Rub}=exp\left[\beta^{Rub}{\left(X_{1}^{Rub}\right)}^{2}\right]$$

where $\beta^{Rub}$ and $X_{1}^{Rub}$ are the interaction parameter and micellar mole fraction, respectively of component 1. If two variables have values of less than 1, the mixing components are not ideal. When computing the $\beta^{Rub}$ values (parameter based on the *cmc* values of each amphiphile and their mixtures), the nature and strength of the interactions between the two surfactants are determined. Rubingh [52] derived the relationship shown in Equation (5) by considering the phase separation model for micellization.

(5) $$\beta^{Rub}=\frac{\ln\left(\alpha_{1}cmc/X_{1}^{Rub}cmc_{1}\right)}{{\left(1-X_{1}^{Rub}\right)}^{2}}\;$$

The micellar mole fraction of component 1 is represented by $X_{1}^{Rub}$, which is calculated by iteratively solving Equation (6):

(6) $$\frac{{\left(X_{1}^{Rub}\right)}^{2}\ln\left(\alpha_{1}cmc/X_{1}^{Rub}cmc_{1}\right)}{{\left(1-X_{1}^{Rub}\right)}^{2}\ln\left[\left(1-\alpha_{1}\right)cmc/\left(1-X_{1}^{Rub}\right)cmc_{2}\right]}=1$$

It is commonly believed that the deviation from zero of the interaction parameters ($\beta^{Rub}$) is due to interactions among the amphiphile head groups. Positive divergence from zero indicates antagonistic behavior, and negative deviation indicates synergistic interactions between two components. Free energy subsidies associated with amphiphile head groups have been found to be the main sources of mutual interaction. When positively and negatively charged amphiphiles are assorted in water, the most noteworthy feature of this mixture is its unusually huge drop in cmc values. A mixture of anionic and non-ionic surfactants usually yields a nonconformity from ideal behavior (less negative $\beta^{Rub}$) and synergistic effects in the mixed micelles of two non-ionic amphiphiles are even to a lesser extent. In most cases, experimentally computed values of $\beta^{Rub}$ for mixtures of positively and negatively charged amphiphiles are higher. According to Table 1, there were considerable interactions (synergism) between the current mixed systems. The synergism was detected because of the electrostatic interaction among +ve and –ve charged head groups. The $\beta^{Rub}$ average values were – 15.90 and – 11.25 for SDS + TCH and SLS + TCH, respectively. The positive and negatively charged amphiphiles were found to be firmly tied to one another through electrostatic and hydrophobic forces, consequently leading to ultimate attraction that promoted the growth of micellar aggregates. The synergism between two amphiphiles depends not only on the strength of the interaction but also on the individual amphiphile properties. The higher the hydrophobicity of an amphiphile, the easier it is to make micelles.

In a mixed system, the ideal micellar mole fraction of component 1 is represented by Equation (7) [54]

(7) $$X_{1}^{ideal}=\frac{\alpha_{1}cmc_{2}}{\alpha_{1}cmc_{2}+\alpha_{2}cmc_{1}}$$

The values of $X_{1}^{ideal}$ are given in Table 1. The values of $X_{1}^{ideal}$ display nonconformity from the values of $X_{1}^{Rub}$, signifying non-ideality. The higher values of $X_{1}^{ideal}$ for both binary mixtures at all mole fractions confirmed that added drug molecules replace some of the surfactant molecules from the mixed micelles, so the contribution of drug molecules is greater in mixed micelles than it should be in ideally mixed systems.

#### Thermodynamic Parameters for Drug–Surfactant Mixtures in the Mixed Micelle

Using RST, it is feasible to evaluate the free energy change for micellization in the following way [55,56,57,58,59]:

(8) $$\Delta G_{mix}=RT[X_{1}^{Rub}ln(X_{1}^{Rub}f_{1}^{Rub})+X_{2}^{Rub}ln\left(X_{2}^{Rub}f_{2}^{Rub}\right)]$$

If the values of activity coefficients ($f_{1}^{Rub}$ and $f_{2}^{Rub}$) for an ideal mixed system are equal to unity, then Equation (8) becomes:

(9) $$\Delta G_{mix}^{ideal}=RT[X_{1}^{Rub}lnX_{1}^{Rub}+X_{2}^{Rub}lnX_{2}^{Rub}]\;$$

where $\Delta G_{mix}^{ideal}$ is the free energy change for an ideal mixed system. Interestingly, the data (Table 2) show that the values were negative, implying that the micelles were spontaneously formed and were stable. If the values of $\Delta G_{mix}^{ideal}$ deviate from the values of $\Delta G_{mix}$, rather than forming an ideal micelle, it then forms a real one. The literature confirms that previous investigators have observed the same behavior [60,61].

An excess thermodynamic function is a variation among the energetic function of the mixer for a non-ideal solution and the subsequent values for an ideal solution at a similar pressure and temperature [54]. The excess free energy of mixed micellization $G_{mix}^{E}$ for a two-amphiphile mixtures can be computed with the help of equations 8 and 9 in form of Equation (10).

(10) $$G_{mix}^{E}=\Delta H_{m}=RT[X_{1}^{Rub}lnf_{1}^{Rub}+X_{2}^{Rub}lnf_{2}^{Rub}]\;$$

From Table 2, we can observe that the values of $G_{mix}^{E}$ were negative over the entire mole fraction range, confirming observations that the creation of the mixed micelles was thermodynamically more stable than the ideal state.

For the mixed system, Equations (9) and (10) were also used to calculate the entropy change as Equation (11):

(11) $$\Delta S_{m}=\frac{\Delta H_{m}-\Delta G_{m}}{T}=-R\left[X_{1}^{Rub}lnX_{1}^{Rub}+X_{2}^{Rub}lnX_{2}^{Rub}\right]$$

Moreover, both binary and mixed micellization were found to be constrained by positive entropy values, which confirmed that entropy contribution drives mixed micellization. In the literature, the same results have previously been reported [55]. When we consider SDS + TCH mixed systems, the contributions to entropy were more significant at initial fractions. It was found to be an entropically favorable process when mixed micelles were formed, as the entropy/free energy change in this process was greater than 0.

Equation (12) was utilized to compute standard Gibbs free energy per mole of micellization using the mass-action model without considering counterion binding [58]:

(12) $$\Delta G_{m}^{o}=RTlnX_{CMC}\;$$

In the above equation, $X_{CMC}$ is the cmc value at mole fraction unit while *R* and *T* have their basic scientific meaning. The values of $\Delta G_{m}^{o}$ listed in Table 2 are negative for single and mixed amphiphiles. The negative values show that the micellization spontaneously occurred in the aqueous NaCl solution. The $\Delta G_{m}^{o}$ values of the drug were less than the single surfactants (SDS or SLS) and mixtures, confirming that mixed micelle formation of a drug with surfactants is more spontaneous compared to a drug alone. It is interesting to note here that the $\beta^{Rub}$ values and $\Delta G_{m}^{o}$ values were directly proportional with respect to $\alpha_{1}$, confirming that the higher interactions between amphiphile monomers cause more spontaneity in the process; the same results were reported by Bagheri et al. [54].

### 2.2. Interfacial Properties of TCH + SDS/SLS Mixed System

When amphiphiles are dissolved in water, the amphiphile monomers are adsorbed at the surface and the surface tension of water decreases, mainly due to the hydrophobic effects. The thermal motion and dynamic equilibrium determine the adsorption or desorption of monomers. Electrostatic interactions, hydrogen bonding, van der Waals interactions, and solvation/desolvation are factors that are less responsible for adsorption. Gibb’s adsorption equation can be used to quantify the amount of amphiphiles adsorbed per unit area of the interface (surface excess, $\Gamma_{max}$) [62]:

(13) $$\Gamma_{\max}=-\frac{1}{2.303nRT}\left(\frac{d\gamma }{dlogC}\right)$$

In Equation (13), $\frac{d\gamma }{dlogC}$ is the maximum slope, T is the absolute temperature in K, and *R* = 8.314 J mol^–1^ K^–1^. Based on literature, the value of *n* was taken as 2 for pure amphiphiles and was calculated for mixtures with the following expression [62,63]

(14) $$n=X_{1}^{s}n_{1}X_{2}^{s}n_{2}\;$$

The $\Gamma_{\max}$ values can be used to calculate the values of minimum area per molecule ($A_{min}$) with Equation (15) [64]

(15) $$A_{min}=\frac{10^{20}}{N_{A}\Gamma_{\max}}$$

where *N_A_* = 6.02214 × 10^23^ (Avogadro’s number). The minimum area per molecule of an amphiphile suggests the packing (loose or close) and orientation of the amphiphile molecule at the surface. The low $A_{min}$ (high $\Gamma_{\max}$) values of the mixture at all mole fractions confirmed strong electrostatic interactions between cationic drugs and anionic surfactants (Table 3). This fact was also reflected in the negative interaction parameter values for the mixture. If there is no interaction between two amphiphiles in a mixed adsorbed film at the surface, the minimum area per molecule can be calculated with the following equation [62]:

(16) $$A_{ideal}=\alpha_{1}A_{min,\;1}+\alpha_{2}A_{min,\;2}$$

The observed values ($A_{min}$) were lower than ideal values ($A_{ideal}$*),* indicating significant attractive interactions between the two components (Table 3). Water became 84–99% saturated following the adsorption of amphiphiles, which reduced its surface tension by approximately 20 dyn/cm. Adding an amphiphile to the water decreased the surface tension of H_2_O by 20 mNm^−1^, indicating the efficiency of its adsorption. Hence, it has the lowest concentration required to achieve saturation adsorption. By using Equation (17), we could calculate the adsorption efficiency ($pC_{20})$ as:

(17) $$pC_{20}=-logC_{20}$$

where *C*_20_ is a measure of the adsorption efficiency of surfactants at the interface. The values of *C*_20_ are also listed in Table 3. It was concluded that the *C*_20_ values of SDS decreased with the addition of TCH. Decreasing *C*_20_ values of SDS with TCH were also shown by an earlier study [51]. In the case of SLS, the values of *C*_20_ only decreased at higher mole fractions. The *C*_20_ value of SDS in the presence NaCl has been found to be lower than in its absence [51], confirming that the surface activity of SDS is enhanced in the presence of NaCl.

Rosen and Hua modified Equations (5) and (6) for amphiphile adsorption to calculate the $X_{1}^{S}$ and $\beta^{s}$ with the following equations [64]

(18) $$\frac{{\left(X_{1}^{s}\right)}^{2}\ln\left(\alpha_{1}C_{mix}/X_{1}^{s}C_{1}\right)}{{\left(1-X_{1}^{S}\right)}^{2}\ln\left[\left(1-\alpha_{1}\right)C_{mix}/\left(1-X_{1}^{S}\right)C_{2}\right]}=1$$

(19) $$\beta^{s}=\frac{ln\left(\alpha_{1}C_{mix}/X_{1}^{S}C_{1}\right)}{{\left(1-X_{1}^{S}\right)}^{2}}$$

The interpretation of interaction parameter at the surface ($\beta^{s})$ is the same as in the case of bulk ($\beta^{Rub})$, with negative and positive $\beta^{s}$ values that suggest synergism and antagonism, respectively. Here, the values of $X_{1}^{s}$ were increased with the stoichiometric mole fraction (Table 4) and were always greater than $X_{1}^{Rub}$, showing amphiphiles contributed more to mixed monolayer formation than in the mixed micelle. Additionally, the contribution of SDS was greater than SLS in the mixed monolayer formation with the TCH. The $\beta^{s}$ values were negative for both mixed systems, suggesting attractive interaction. The activity coefficients at the surface could be calculated by the following equations

(20) $$lnf_{1}^{S}=\beta^{s}{\left(X_{2}^{S}\right)}^{2}$$

(21) $$lnf_{2}^{S}=\beta^{s}{\left(X_{1}^{S}\right)}^{2}$$

The values of $f_{1}^{S}$ and $f_{2}^{S}$ are listed in Table 4 and were found to be less than unity, thus indicating non-ideality at the surface.

#### Thermodynamic Parameters for Drug–Surfactant Mixtures at the Surface

The standard free energy of interfacial adsorption ($\Delta G_{add}^{o}$) can be computed by using the following relation [58]:

(22) $$\Delta G_{add}^{o}=\Delta G_{m}^{o}-\left(\frac{\pi_{CMC}}{\Gamma_{max}}\right)\;$$

At the cmc, surface pressure is measured with the term $\pi_{CMC}$. Here in Equation (22), $G_{m}^{o}$ is the standard Gibbs free energy previously computed with Equation (12). It was observed that the accomplished upsides of $\Delta G_{add}^{o}$ were –ve, similar to those of $\Delta G_{m}^{o}$; nonetheless, the extent was much more noteworthy, showing that adsorption was further unconstrained for this situation. $f_{1}^{S}$ and $f_{2}^{S}$ can be utilized to ascertain excess free energy ($G_{exc}^{s}$) at surface:

(23) $$G_{exc}^{s}=RT\left[X_{1}^{S}lnf_{1}^{S}+\left(1-X_{1}^{S}\right)lnf_{2}^{S}\right]$$

With negative values of $G_{exc}^{s}$, stability can be attained by the stable mixing at the surface, which is possible with the monolayer of surfactants or drugs alone. Negative $G_{exc}^{s}$ values (Table 4) also indicate synergism at the surface. The degree of synergism for a mixed system can also be quantified by an energy parameter [65],

(24) $$G_{min}^{s}=A_{min}\gamma_{CMC}N_{A}$$

The energy parameters that define the work required to create an interface per mole of the solution by transferring monomers from bulk to interface can be determined by the above-described energy parameters ($G_{min}^{s}$). According to Table 4, a lower value of $G_{min}^{s}$ indicates a more stable surface, and this in turn results in increased surface activity.

## 3. UV–Visible Spectroscopic Study

**Figure 5:**
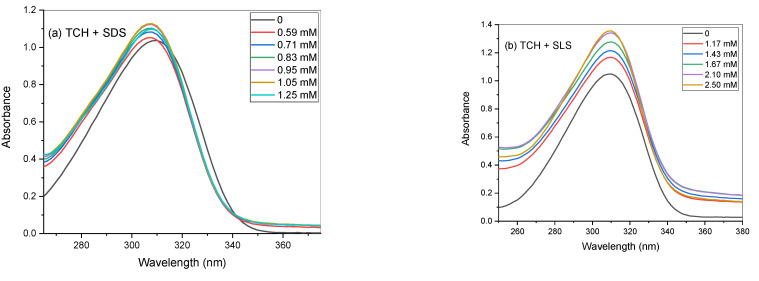
Absorption spectra of tetracaine hydrochloride in the presence of increased concentrations of (**a**) TCH + SDS and (**b**) TCH + SLS.

The interaction of TCH with SDS and SLS was monitored with UV–visible absorption spectroscopy. The absorption spectrum of TCH (0.05 mM) in a 100 mM NaCl solution showed two absorption peaks at 226 and 310 nm due to the attendance of the aminobenzoate group. π–π* and *n*–π* transitions were involved in the first and second ones, respectively. When increasing concentrations of SDS and SLS were added to the TCH solution, the absorbance increased but the maximum absorbance at 310 nm was not changed (Figure 5). This spectral behavior indicates the electrostatic interactions between the positive charge of TCH molecules and the negative charge of surfactant monomers.

**Figure 6:**
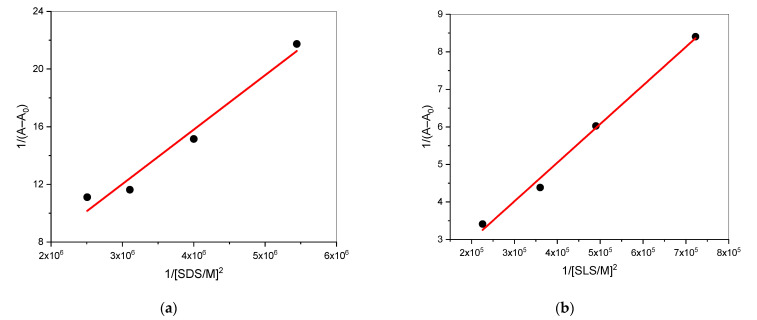
Benesi–Hildebrand plots for the interaction of TCH (**a**) SDS and (**b**) SLS.

The binding constant and stoichiometric ratio were estimated with the differential absorbance method represented by the Benesi–Hildebrand equation [66]:

(25) $$\frac{1}{A-A_{0}}=\frac{1}{K\left(A_{max}-A_{0}\right){\left[S\right]}^{n}}+\frac{1}{A_{max}-A_{0}}$$

where the concentration of SDS/SLS is represented by [S], while *A*, *A*_0_, and *A*_max_ represent values of absorbance due to the presence of surfactants, the absence of surfactants, and resulting absorbance due to the drug–surfactant complex, respectively. When plotting 1/(*A − A_0_*) against 1/[SDS/SLS]^2^, a straight line is obtained (Figure 6), specifying the creation of the 1:2 complex. For an SDS + TCH mixed system without the addition of salt, Thapa et. al. reported a 1:1 complex [51]. However, for our system, a curvilinear fit was obtained, so the SDS + TCH complex was mainly 1:2. Using the Benesi–Hildebrand equation, the binding constant could be calculated (intercept/slope). We found values of K of 1.86 × 10^5^ (± 0.04) and 9.09 × 10^4^ (± 0.04) mol^–1^ dm^3^ for the SDS + TCH and SLS + TCH mixed systems, respectively. The SLS + TCH mixed system had lesser binding constant values than the SDS + TCH system. In comparison, SDS has one functional group and SLS has two functional groups. The localized positive charge on the nitrogen atom on the TCH interacts with the negative charge on the sulphonic group, thus enhancing the electrostatic attraction between the guest and host. SLS, however, has methylated amide nitrogen, so the amide bond cannot be a hydrogen bond donor, which inhibits intermolecular attraction between SLS and TCH at the palisade layer. Furthermore, the steric hindrance of the N-methyl group of SLS may make it difficult to tightly align the amphiphiles. All these behaviors of SLS are responsible for its lesser binding constant compared to SDS.

By using binding constant (*K*) values, free energy change of binding could be attained with Equation (26):

(26) $$\Delta G_{K}=-RTlnK$$

The binding free energies were –30.08 (± 0.2) Jmol^–1^ for SDS + TCH and – 28.30(± 0.2) kJmol^–1^ for SLS + TCH. In both mixed systems, the G values were negative, indicating that the binding process was spontaneous.

## 4. Conclusions

The synergistic interaction of TCH (+ve charged head group) with SDS and SLS (–ve charged head group) surfactants in the presence of salt (100 mM NaCl) was analyzed with both tensiometry and UV–visible spectroscopic techniques. The following conclusions can be derived:

1. The negative deviation of experimentally determined cmc values with hypothetical values confirms the nonideality of current mixtures.
2. The interaction parameter at the interface and in solution was determined to be –ve, thus validating synergism between monomers of two species at the surface and in bulk.
3. The higher values of the ideal mole fraction of component 1 ($X_{1}^{ideal}$) for both binary mixtures at all mole fractions indicate the strong ability of the drug to form of mixed micelles.
4. Energetics parameters confirm the spontaneity, stability, and entropic favorability of drug–surfactant mixtures.
5. The TCH with SLS had smaller binding constant values than SDS, possibly because SLS has a methylated amide nitrogen so the amide bond cannot be a hydrogen bond donor, which inhibits the intermolecular attraction between SLS and TCH at the palisade layer. Furthermore, the steric hindrance of the N-methyl group of SLS may make it difficult to tightly align the amphiphiles. All these behaviors of SLS are responsible for its smaller binding constant in comparison to SDS.

## 5. Experimental

### 5.1. Materials

Tetracaine hydrochloride (TCH, 99%), an anesthetic amphiphilic drug, and sodium lauroyl sarcosine (SLS, >95%) were supplied by Molecules On (Switzerland) and used as received. Sodium chloride (NaCl, 99%) and sodium dodecyl sulfate (SDS, 98.5%) were acquired from Sigma-Aldrich (St. Louis, MO, USA). At 298.15 K, all experiments were performed using ultra-pure, double-distilled de-ionized water with a conductivity between 1 and 2 µScm^–1^. To prepare standard solutions for experiments, amphiphiles (both pure and mixed) were dissolved and accurately weighed in a 100 mM NaCl solution. The stock solutions for both techniques (surface tension and UV–vis spectrophotometer measurements) were prepared in aqueous 100 mM NaCl solutions.

### 5.2. Methods

#### 5.2.1. Surface Tension Measurements

The surface tension experiments were conducted with a digital tensiometer (Sigma 700, Attention, Darmstadt, Germany) by using a platinum ring. The instrument was occasionally calibrated with ultra-pure distilled water. In tensiometric titration, an amphiphile stock solution was titrated into a static volume of H_2_O. Throughout all experiments, water was circulated from a thermostatically controlled water bath through the outer jacket to keep the temperature at 298.15 K.

#### 5.2.2. UV–Vis Spectrophotometer Measurements

We measured the spectra of the aqueous solutions of the drug and the drug–surfactant binary mixtures to determine the level of the binding of the drug with surfactants. As a first step, TCH in water was prepared as a stock solution in a volumetric flask. The desired concentration of surfactant solution was prepared from the aqueous TCH solution. Finally, a suitable volume of surfactant solution was added to the H_2_O solution of TCH in a quartz cell. We measured the absorption spectra of TCH solutions with surfactants and plotted them against the wavelengths. For the measurement of the absorption spectrum of TCH solutions over the range of 200–400 nm, an Evolution 300 spectrophotometer from Thermo Scientific, Tokyo, Japan was used to record UV–visible spectra (Figure 2).

## Figures and Tables

**Table 1:** Physical parameters of TCH + SDS/SLS mixed systems in aqueous NaCl.

	*cmc* (mM)	cmc* (mM)	$X_{1}^{Rub}$	$X_{1}^{ideal}$	−*β^Rub^*	$f_{1}^{Rub}$	$f_{2}^{Rub}$
SDS + TCH							
0.0	79.43	-	-	-	-	-	-
0.05	0.37	16.42	0.54	0.80	16.04	0.033	0.0095
0.1	0.31	9.16	0.56	0.90	15.20	0.055	0.0081
0.7	0.13	1.45	0.65	0.99	15.72	0.139	0.0014
0.8	0.15	1.27	0.66	0.99	15.44	0.174	0.0011
0.9	0.12	1.13	0.67	0.99	17.11	0.156	0.0004
1.0	1.02	-	-	-	-	-	-
SLS + TCH							
0.0	79.43	-	-	-	-	-	-
0.05	4.49	41.24	0.50	0.51	8.86	0.110	0.1078
0.1	3.05	27.85	0.53	0.68	9.09	0.139	0.0742
0.5	1.24	7.74	0.62	0.95	9.96	0.243	0.0207
0.7	0.79	5.68	0.64	0.98	11.81	0.212	0.0082
0.9	0.33	4.49	0.64	0.99	16.53	0.115	0.0012
1.0	4.07	-	-	-	-	-	-

Relative standard uncertainties (*u_r_*) are *u_r_*(cmc/cmc*) = 0.03, *u_r_* ($X_{1}^{Rub}$/$X_{1}^{ideal}$) = 0.02, *u_r_* (*β^Ru^*) = 0.03, and *u_r_* ($f_{1}^{Rub}$/ $f_{2}^{Rub}$) = 0.04.

**Table 2:** Energetic constraints of TCH + SDS/SLS mixtures in aqueous NaCl ^a^.

α_1_	$\text{−}G_{mix}^{E}/-\Delta H_{m}\;({\mathrm{kJmol}}^{-1})$	$-\Delta G_{mix}\;({\mathrm{kJmol}}^{-1})$	$-\Delta G_{mix}^{ideal}\;({\mathrm{kJmol}}^{-1})$	$T\Delta S_{m}\;({\mathrm{kJmol}}^{-1})$	$\left|\frac{T\Delta S_{m}}{\Delta G_{mix}}\right|$	$-\Delta G_{m}^{o}\;({\mathrm{kJmol}}^{-1})$
SDS + TCH						
0.0	-	-	-	-	-	16.23
0.05	9.87	11.78	1.71	6.40	0.54	29.48
0.1	9.27	11.15	1.69	6.32	0.57	29.93
0.7	8.90	10.69	1.61	6.01	0.56	32.08
0.8	8.54	10.29	1.58	5.88	0.57	31.79
0.9	9.36	11.12	1.57	5.89	0.53	32.34
1.0	-	-	-	-	-	27.01
SLS + TCH						
0.0	-	-	-	-	-	16.23
0.05	5.49	7.33	1.72	6.17	0.84	23.34
0.1	5.60	7.44	1.71	6.16	0.82	24.30
0.5	5.79	7.56	1.64	5.93	0.78	26.54
0.7	6.76	8.52	1.62	5.92	0.69	27.66
0.9	9.45	11.26	1.62	6.07	0.54	29.77
1.0	-	-	-	-	-	23.59

^a^ Relative standard uncertainties (*u_r_*) are *u_r_*($G_{mix}^{E}$/$\Delta H_{m}$) = 0.03, *u_r_*($\Delta G_{mix}/\Delta G_{mix}^{ideal}$) = 0.03, *u_r_*($\Delta S_{m}$) = 0.03, and *u_r_*($\Delta G_{m}^{o}$) = 0.03.

**Table 3:** Interfacial and packing data of TCH + SDS/SLS mixed system in aqueous NaCl ^a^.

α_1_	10^6^ Γ_max_ (molm^−2^)	*A_min_* (Å^2^)	*A_ideal_* (Å^2^)	*C*_*20*_	*γ*_*cmc*_ (mNm^–1^)	*π*_*cmc*_ (mNm^–1^)
SDS + TCH						
0.0	1.64	1.01	-	19.36	39.57	31.43
0.05	1.77	0.94	1.01	0.03	27.79	43.21
0.1	2.44	0.68	1.01	0.05	28.55	42.45
0.7	3.10	0.53	0.99	0.03	29.88	41.12
0.8	3.39	0.49	0.98	0.04	30.17	40.83
0.9	3.28	0.51	0.97	0.03	30.68	40.32
1.0	1.71	0.97	-	0.09	30.60	40.40
SLS + TCH						
0.0	1.64	1.01	-	19.36	39.57	31.43
0.05	2.73	0.61	1.01	0.80	27.88	43.11
0.1	2.28	0.73	1.01	0.41	27.72	43.28
0.5	2.57	0.65	1.03	0.19	26.89	44.11
0.7	2.14	0.77	1.04	0.09	27.11	43.89
0.9	2.01	0.83	1.05	0.04	28.04	42.96
1.0	1.57	1.05	-	0.18	23.80	47.20

^a^ Relative standard uncertainties (*u_r_*) are *u_r_*(Γ_max_) = 0.05, *u_r_*(*A_min_/A_ideal_*) = 0.03, *u_r_*(*C_20_*) = 0.03, and *u_r_*(***γ_cmc_****/π_cmc_*) = 0.02.

**Table 4:** Thermodynamic and interfacial properties of TCH + SDS/SLS mixtures in aqueous NaCl ^a^.

α_1_	$X_{1}^{s}$	$-\beta^{s}$	$f_{1}^{s}$	$f_{2}^{s}$	$-G_{ex}^{s}\;({\mathrm{kJmol}}^{-1})$	–Δ*G_ads_* (kJmol^−1^)	*G_min_* (kJmol^−1^)
	SDS + TCH						
0.0	-	-	-	-	-	35.34	24.07
0.05	0.56	17.66	0.033	0.004	10.77	53.89	15.69
0.1	0.59	14.60	0.091	0.006	8.71	47.35	11.71
0.7	0.71	12.28	0.370	0.002	6.19	45.33	9.62
0.8	0.74	11.40	0.486	0.002	5.32	43.82	8.88
0.9	0.75	12.83	0.454	0.001	5.92	44.61	9.34
1.0	-	-	-	-	-	50.74	17.97
	SLS + TCH						
0.0	-	-	-	-	-	34.83	24.07
0.05	0.54	19.05	0.018	0.004	11.71	39.16	10.22
0.1	0.57	16.09	0.051	0.005	9.77	43.27	12.14
0.5	0.64	14.52	0.157	0.002	8.25	43.71	10.47
0.7	0.67	13.48	0.251	0.001	7.26	48.17	12.67
0.9	0.70	15.10	0.261	0.001	7.83	51.15	13.94
1.0		-	-	-	-	53.58	15.12

^a^ Relative standard uncertainties (*u_r_*) are *u_r_*($X_{1}^{S}$) = 0.02, *u_r_*(*β^s^*) = 0.03, *u_r_*($f_{1}^{s}/f_{2}^{s}$) = 0.04, *u_r_*($G_{ex}^{s}$) = 0.03, *u_r_*(Δ*G_ads_*) = 0.03, and *u_r_*(*G_min_*) = 0.03.

## Data Availability

Not applicable.

## References

[1] Van Eerdenbrugh B., Vermant J., Martens J.A., Froyen L., Van Humbeeck J., Van den Mooter G., Augustijns P. (2010). Solubility Increases Associated with Crystalline Drug Nanoparticles: Methodologies and Significance. Mol. Pharm..

[2] Ruso J.M., Attwood D., Rey C., Taboada P., Mosquera V., Sarmiento F. (1999). Light Scattering and NMR Studies of the Self-Association of the Amphiphilic Molecule Propranolol Hydrochloride in Aqueous Electrolyte Solutions. J. Phys. Chem. B.

[3] Awang N., Ismail A.F., Jaafar J., Matsuura T., Junoh H., Othman M.H.D., Rahman M.A. (2015). Functionalization of polymeric materials as a high performance membrane for direct methanol fuel cell: A review. React. Funct. Polym..

[4] Azum N., Rub M.A., Khan A., Alotaibi M.M., Asiri A.M., Rahman M.M. (2022). Mixed Micellization, Thermodynamic and Adsorption Behavior of Tetracaine Hydrochloride in the Presence of Cationic Gemini/Conventional Surfactants. Gels.

[5] Rub M.A., Azum N., Kumar D., Asiri A.M. (2022). Interaction of TX-100 and Antidepressant Imipramine Hydrochloride Drug Mixture: Surface Tension, 1H NMR, and FT-IR Investigation. Gels.

[6] Ahmed M.F., Abdul Rub M., Joy M.T.R., Molla M.R., Azum N., Anamul Hoque M., Rub M.A., Azum N., Kumar D., Asiri A.M. (2022). Influences of NaCl and Na2SO4 on the Micellization Behavior of the Mixture of Cetylpyridinium Chloride + Polyvinyl Pyrrolidone at Several Temperatures. Gels.

[7] Alvarez-Lorenzo C., Concheiro A. (2003). Effects of Surfactants on Gel Behavior. Am. J. Drug Deliv..

[8] Wedel B., Brändel T., Bookhold J., Hellweg T. (2017). Role of Anionic Surfactants in the Synthesis of Smart Microgels Based on Different Acrylamides. ACS Omega.

[9] Maiti K., Mitra D., Mitra R.N., Panda A.K., Das P.K., Rakshit A.K., Moulik S.P. (2010). Self-Aggregation of Synthesized Novel Bolaforms and Their Mixtures with Sodium Dodecyl Sulfate (SDS) and Cetyltrimethylammonium Bromide (CTAB) in Aqueous Medium. J. Phys. Chem. B.

[10] Jafari-Chashmi P., Bagheri A. (2018). The strong synergistic interaction between surface active ionic liquid and anionic surfactant in the mixed micelle using the spectrophotometric method. J. Mol. Liq..

[11] Mal A., Bag S., Ghosh S., Moulik S.P. (2018). Physicochemistry of CTAB-SDS interacted catanionic micelle-vesicle forming system: An extended exploration. Colloids Surf. A Physicochem. Eng. Asp..

[12] Tozuka Y., Imono M., Uchiyama H., Takeuchi H. (2011). A novel application of α-glucosyl hesperidin for nanoparticle formation of active pharmaceutical ingredients by dry grinding. Eur. J. Pharm. Biopharm..

[13] Shen S., Ng W.K., Chia L., Dong Y., Tan R.B.H. (2010). Stabilized Amorphous State of Ibuprofen by Co-Spray Drying With Mesoporous SBA-15 to Enhance Dissolution Properties. J. Pharm. Sci..

[14] Sigfridsson K., Lundqvist A.J., Strimfors M. (2009). Particle size reduction for improvement of oral absorption of the poorly soluble drug UG558 in rats during early development. Drug Dev. Ind. Pharm..

[15] Sugano K., Okazaki A., Sugimoto S., Tavornvipas S., Omura A., Mano T. (2007). Solubility and Dissolution Profile Assessment in Drug Discovery. Drug Metab. Pharmacokinet..

[16] Schreier S., Malheiros S.V.P., de Paula E. (2000). Surface active drugs: Self-association and interaction with membranes and surfactants. Physicochemical and biological aspects. Biochim. Et Biophys. Acta (BBA)-Biomembr..

[17] YOKOYAMA S., FUJINO Y., KAWAMOTO Y., KANEKO A., FUJIE T. (1994). Micellization of an Aqueous Solution of Piperidolate Hydrochloride in the Presence of Acetylcholine Chloride. Chem. Pharm. Bull..

[18] Attwood D., Tolley J.A. (2011). Self-association of analgesics in aqueous solution: Association models for codeine, oxycodone, ethylmorphine and pethidine. J. Pharm. Pharmacol..

[19] Kumar D., Azum N., Rub M.A., Asiri A.M. (2021). Interfacial and spectroscopic behavior of phenothiazine drug/bile salt mixture in urea solution. Chem. Pap..

[20] Ghosh S., Krishnan A., Das P.K., Ramakrishnan S. (2003). Determination of Critical Micelle Concentration by Hyper-Rayleigh Scattering. J. Am. Chem. Soc..

[21] Zhu Q., Huang L., Su J., Liu S. (2014). A sensitive and visible fluorescence-turn-on probe for the CMC determination of ionic surfactants. Chem. Commun..

[22] Tadros T.F. (2005). Applied Surfactants.

[23] Chiu Y.C., Kuo C.Y., Wang C.W. (2000). Using electrophoresis to determine zeta potential of micelles and critical micelle concentration. J. Dispers. Sci. Technol..

[24] Priev A., Zalipsky S., Cohen R., Barenholz Y. (2002). Determination of Critical Micelle Concentration of Lipopolymers and Other Amphiphiles: Comparison of Sound Velocity and Fluorescent Measurements. Langmuir.

[25] Romani A.P., da Hora Machado A.E., Hioka N., Severino D., Baptista M.S., Codognoto L., Rodrigues M.R., de Oliveira H.P.M. (2009). Spectrofluorimetric Determination of Second Critical Micellar Concentration of SDS and SDS/Brij 30 Systems. J. Fluoresc..

[26] Pérez-Rodríguez M., Prieto G., Rega C., Varela L.M., Sarmiento F., Mosquera V. (1998). A Comparative Study of the Determination of the Critical Micelle Concentration by Conductivity and Dielectric Constant Measurements. Langmuir.

[27] Karsa D.R. (1999). Industrial Applications of Surfactants.

[28] Atta A.M., Abdullah M.M.S., Al-Lohedan H.A., Ezzat A.O. (2018). Demulsification of heavy crude oil using new nonionic cardanol surfactants. J. Mol. Liq..

[29] Shaban S.M., Kang J., Kim D.-H. (2020). Surfactants: Recent advances and their applications. Compos. Commun..

[30] Hegazy M.A., Abdallah M., Ahmed H. (2010). Novel cationic gemini surfactants as corrosion inhibitors for carbon steel pipelines. Corros. Sci..

[31] Torchilin V.P. (2001). Structure and design of polymeric surfactant-based drug delivery systems. J. Control. Release.

[32] King S.-Y.P., Basista A.M., Torosian G. (1989). Self-Association and Solubility Behaviors of a Novel Anticancer Agent, Brequinar Sodium. J. Pharm. Sci..

[33] Matsuki H., Hashimoto S., Kaneshina S., Yamanaka M. (1994). Surface Adsorption and Volume Behavior of Local Anesthetics. Langmuir.

[34] Atherton A.D., Barry B.W. (2011). Photon correlation spectroscopy of surface active cationic drugs. J. Pharm. Pharmacol..

[35] Sarmiento F., López-Fontán J.L., Prieto G., Mosquera V., Attwood D. (1997). Mixed micelles of structurally related antidepressant drugs. Colloid Polym. Sci..

[36] Rub M.A., Azum N., Khan F., Asiri A.M. (2017). Surface, micellar, and thermodynamic properties of antidepressant drug nortriptyline hydrochloride with TX-114 in aqueous/urea solutions. J. Phys. Org. Chem..

[37] Abdul Rub M., Azum N., Asiri A.M. (2017). Binary Mixtures of Sodium Salt of Ibuprofen and Selected Bile Salts: Interface, Micellar, Thermodynamic, and Spectroscopic Study. J. Chem. Eng. Data.

[38] Azum N., Naqvi A.Z., Rub M.A., Asiri A.M. (2017). Multi-technique approach towards amphiphilic drug-surfactant interaction: A physicochemical study. J. Mol. Liq..

[39] Azum N., Rub M.A., Asiri A.M., Bawazeer W.A. (2017). Micellar and interfacial properties of amphiphilic drug–non-ionic surfactants mixed systems: Surface tension, fluorescence and UV–vis studies. Colloids Surf. A Physicochem. Eng. Asp..

[40] Kumar D., Rub M.A., Azum N., Asiri A.M. (2018). Mixed micellization study of ibuprofen (sodium salt) and cationic surfactant (conventional as well as gemini). J. Phys. Org. Chem..

[41] Khan F., Rub M.A., Azum N., Asiri A.M. (2018). Mixtures of antidepressant amphiphilic drug imipramine hydrochloride and anionic surfactant: Micellar and thermodynamic investigation. J. Phys. Org. Chem..

[42] Azum N., Rub M.A., Asiri A.M. (2018). Interaction of antipsychotic drug with novel surfactants: Micellization and binding studies. Chin. J. Chem. Eng..

[43] Kumar D., Azum N., Rub M.A., Asiri A.M. (2018). Aggregation behavior of sodium salt of ibuprofen with conventional and gemini surfactant. J. Mol. Liq..

[44] Rub M.A., Azum N., Khan F., Asiri A.M. (2018). Aggregation of sodium salt of ibuprofen and sodium taurocholate mixture in different media: A tensiometry and fluorometry study. J. Chem. Thermodyn..

[45] Azum N., Ahmed A., Rub M.A., Asiri A.M., Alamery S.F. (2019). Investigation of aggregation behavior of ibuprofen sodium drug under the influence of gelatin protein and salt. J. Mol. Liq..

[46] Srivastava A., Thapa U., Saha M., Jalees M. (2019). Aggregation behaviour of tetracaine hydrochloride with Gemini surfactants and the formation of silver nanoparticles using drug-Gemini surfactants mixture. J. Mol. Liq..

[47] Zhou S., Huang G., Chen G. (2019). Synthesis and biological activities of local anesthetics. RSC Adv..

[48] Miller K.J., Goodwin S.R., Westermann-Clark G.B., Shah D.O. (1993). Importance of molecular aggregation in the development of a topical local anesthetic. Langmuir.

[49] Ray G.B., Ghosh S., Moulik S.P. (2009). Physicochemical Studies on the Interfacial and Bulk Behaviors of Sodium N-Dodecanoyl Sarcosinate (SDDS). J. Surfactants Deterg..

[50] Umlong I.M., Ismail K. (2007). Micellization behaviour of sodium dodecyl sulfate in different electrolyte media. Colloids Surf. A Physicochem. Eng. Asp..

[51] Thapa U., Kumar M., Chaudhary R., Singh V., Singh S., Srivastava A. (2021). Binding behaviour of hydrophobic drug tetracaine hydrochloride used as organic counterion on ionic surfactants. J. Mol. Liq..

[52] Holland P.M., Rubingh D.N. (1983). Nonideal multicomponent mixed micelle model. J. Phys. Chem..

[53] Clint J.H. (1975). Micellization of mixed nonionic surface active agents. J. Chem. Soc. Faraday Trans. 1 Phys. Chem. Condens. Phases.

[54] Motomura K., Yamanaka M., Aratono M. (1984). Thermodynamic consideration of the mixed micelle of surfactants. Colloid Polym. Sci..

[55] Negm N.A., Tawfik S.M. (2012). Studies of Monolayer and Mixed Micelle Formation of Anionic and Nonionic Surfactants in the Presence of Adenosine-5-monophosphate. J. Solut. Chem..

[56] Ren Z.H., Huang J., Zheng Y.C., Lai L., Yu X.R., Chang Y.L., Li J.G., Zhang G.H. (2019). Mixed micellization of binary mixture of amino sulfonate amphoteric surfactant with octadecyltrimethyl ammonium bromide in water/isopropanol solution: Comparison with that in aqueous solution. J. Dispers. Sci. Technol..

[57] Das S., Ghosh S., Das B. (2018). Formation of Mixed Micelle in an Aqueous Mixture of a Surface Active Ionic Liquid and a Conventional Surfactant: Experiment and Modeling. J. Chem. Eng. Data.

[58] Rosen M.J., Cohen A.W., Dahanayake M., Hua X.Y. (1982). Relationship of structure to properties in surfactants. 10. Surface and thermodynamic properties of 2-dodecyloxypoly(ethenoxyethanol)s, C12H25(OC2H4)xOH, in aqueous solution. J. Phys. Chem..

[59] Bagheri A., Abolhasani A. (2015). Binary mixtures of cationic surfactants with triton X-100 and the studies of physicochemical parameters of the mixed micelles. Korean J. Chem. Eng..

[60] Ren Z.H., Luo Y., Zheng Y.C., Wang C.J., Shi D.P., Li F.X. (2015). Micellization behavior of the mixtures of amino sulfonate amphoteric surfactant and octadecyltrimethyl ammonium bromide in aqueous solution at 40 °C: A tensiometric study. J. Mater. Sci..

[61] Ren Z.H. (2014). Interacting behavior between amino sulfonate amphoteric surfactant and octylphenol polyoxyethylene ether (7) in aqueous solution and pH effect. J. Ind. Eng. Chem..

[62] Zhou Q., Rosen M.J. (2003). Molecular Interactions of Surfactants in Mixed Monolayers at the Air/Aqueous Solution Interface and in Mixed Micelles in Aqueous Media: The Regular Solution Approach. Langmuir.

[63] Rosen M.J., Hua X.Y. (1982). Surface concentrations and molecular interactions in binary mixtures of surfactants. J. Colloid Interface Sci..

[64] Ananda K., Yadav O.P., Singh P.P. (1991). Studies on the surface and thermodynamic properties of some surfactants in aqueous and water+1,4-dioxane solutions. Colloids Surf..

[65] Oida T., Nakashima N., Nagadome S., Ko J.-S., Oh S.-W., Sugihara G. (2003). Adsorption and Micelle Formation of Mixed Surfactant Systems in Water. III. A Comparison between Cationic Gemini/Cationic and Cationic Gemini/Nonionic Combinations. J. Oleo Sci..

[66] Benesi H.A., Hildebrand J.H. (1949). A Spectrophotometric Investigation of the Interaction of Iodine with Aromatic Hydrocarbons. J. Am. Chem. Soc..

